# Pre-incision traction method using clip-and-thread for gastric endoscopic submucosal dissection

**DOI:** 10.1055/a-2512-4706

**Published:** 2025-01-28

**Authors:** Eisuke Nakao, Satoshi Asai, Ayumu Chaen, Tomoya Hashimura, Yuma Fujita, Eisuke Akamine

**Affiliations:** 1 38434 Department of Gastroenterology, Tane General Hospital, Osaka, Japan

Gastric endoscopic submucosal dissection (G-ESD) has become a widely accepted and well-established treatment modality 1 . However, G-ESD can be technically challenging depending on the lesion’s location owing to the difficulty in creating a mucosal flap. This can increase procedure time and lead to unintentional bleeding during mucosal incision and submucosal dissection. To address these challenges, we present a new method called the “pre-incision traction method” for G-ESD.

A 67-year-old man presented with early gastric cancer located on the greater curvature of the middle gastric body ( Fig. 1 ). A semi-circumferential mucosal incision on the oral side was performed using the ITknife 2 (Olympus, Tokyo, Japan) ( Fig. 2 **a** ). Subsequently, a threaded clip (EZ clip; Olympus) was applied to the anal side where the mucosal incision had not yet been completed ( Fig. 2 **b** ). When mucosal incision on the anal side of the clip was performed with FlushKnife BT-S (Fujifilm, Tokyo, Japan), a mucosal flap was immediately created, providing an excellent field of view and facilitating submucosal dissection ( Fig. 2 **c** ). The dissection speed was also improved due to the adequate tension provided by the clip. The threaded clip did not interfere with subsequent procedures, and a constant tension was kept by pulling the thread. En bloc resection was achieved without any complications ( Fig. 2 **d** , Video 1 ).

**Fig. 1:**
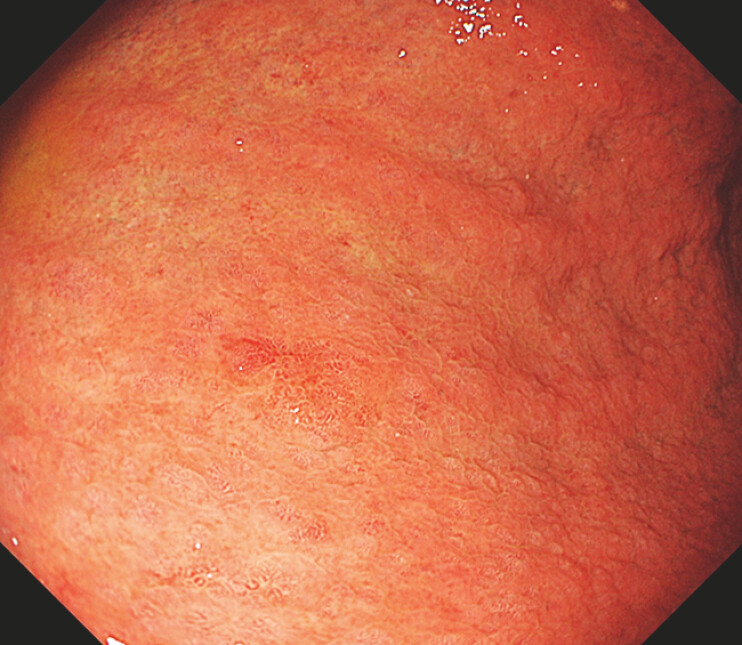
An early gastric cancer located on the greater curvature of the middle gastric body.

**Fig. 2:**
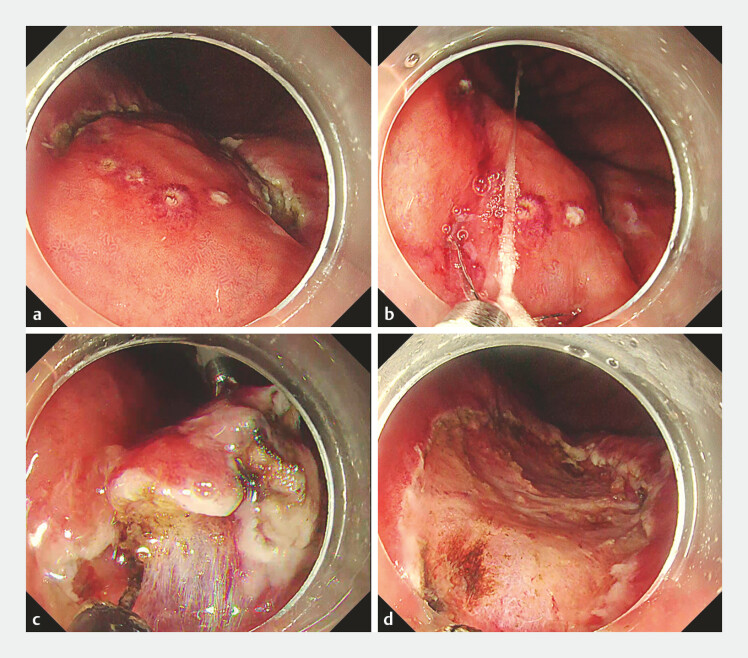
Pre-incision traction method for gastric endoscopic submucosal dissection. **a** A semi-circumferential mucosal incision was performed on the oral side. **b** A threaded clip was applied to the anal side. **c** When mucosal incision on the anal side of the clip was performed, a mucosal flap was immediately created. **d** En bloc resection was achieved without any complications.

Pre-incision traction method using clip-and-thread for gastric endoscopic submucosal dissection.Video 1

The traction methods reported to date typically involve a full circumferential incision to facilitate submucosal dissection 2 . Our novel “pre-incision traction method” aims to simplify the creation of the mucosal flap and maintain a clear field of view, thereby making G-ESD safer and more time efficient. While similar traction methods have been reported for colorectal ESD 3 , there have been no such reports for G-ESD. We are currently planning a feasibility study to investigate the efficacy and safety of this new method.

Endoscopy_UCTN_Code_CPL_1AH_2AZ_3AD

## Notes

Endoscopy E-Videos https://eref.thieme.de/e-videos
*E-Videos* is an open access online section of the journal *Endoscopy* , reporting on interesting cases and new techniques in gastroenterological endoscopy. All papers include a high-quality video and are published with a Creative Commons CC-BY license. Endoscopy E-Videos qualify for HINARI discounts and waivers and eligibility is automatically checked during the submission process. We grant 100% waivers to articles whose corresponding authors are based in Group A countries and 50% waivers to those who are based in Group B countries as classified by Research4Life (see: https://www.research4life.org/access/eligibility/ ). This section has its own submission website at https://mc.manuscriptcentral.com/e-videos .
